# Supplementary material for: Metabolic Reprogramming Induced by Aging Modifies the Tumor Microenvironment
Source: Cells. 2024 Oct 17;13(20):1721. doi: 10.3390/cells13201721 (PMC11506685; doi:10.3390/cells13201721)
Supplement: Supplementary file 1 [file cells-13-01721-s001.zip › cells-3243962-supplementary/Supplenmentary Figures.pdf]

Metabolic reprogramming induced by aging modifies the tumor immune microenvironment

Xingyu Chen[1,†], Zihan Wang[1,†], Bo Zhu[1], Min Deng[2], Jiayue Qiu[1], Yunwen Feng[1], Ning Ding[1], Chen Huang[1,*]

[1]Dr. Neher's Biophysics Laboratory for Innovative Drug Discovery, State Kay Laboratory of Quality Research in Chinese Medicine & Faculty of Chinese Medicine, Macau University of Science and Technology, Taipa, Macao SAR 999078, China
[2]Faculty of Health Sciences, University of Macau, Taipa, Macau SAR 999078, China

Corresponding authors.
E-mail address: chuang@must.edu.mo (C. Huang)
[†]These authors contributed equally to this work

Supplementary Figures

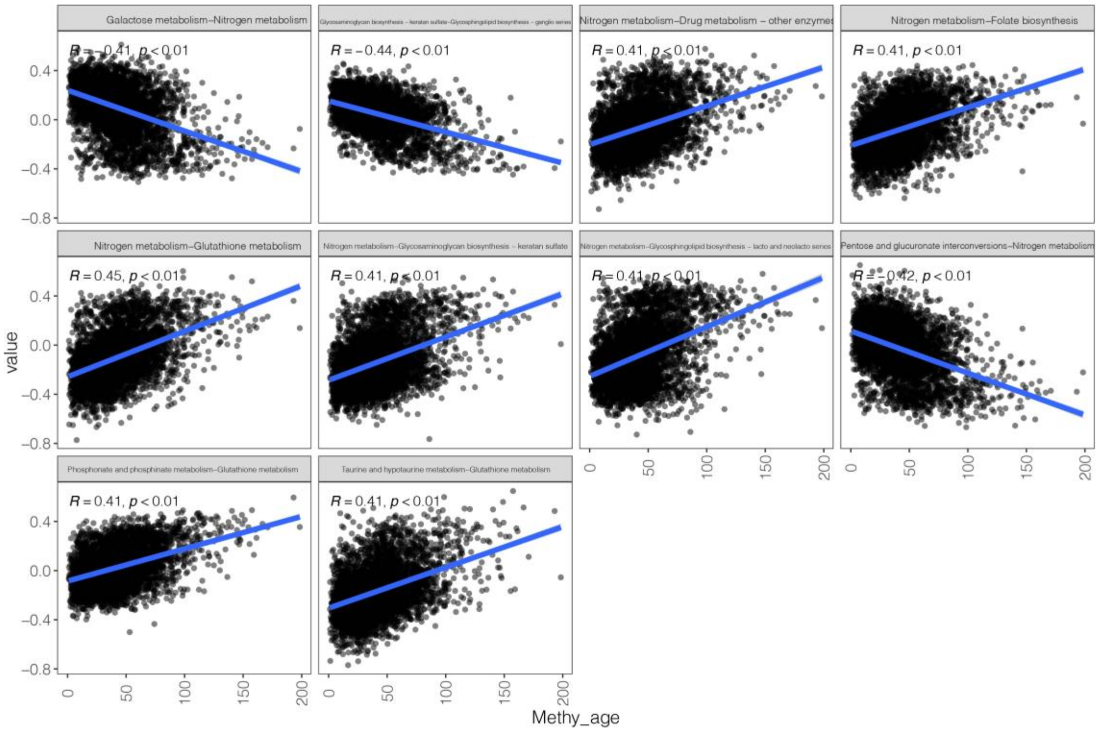

Figure S1. Pearson correlation analysis between methylation age and metabolic pathway pair activity score.

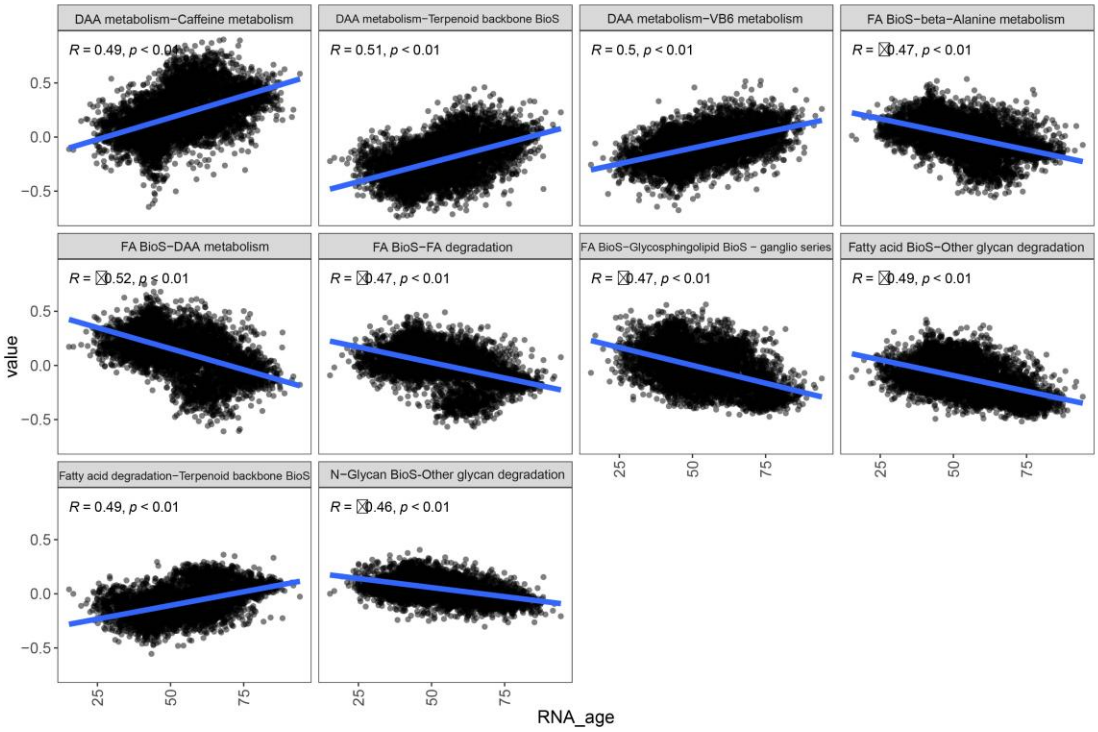

Figure S2. Pearson correlation analysis between RNA age and metabolic pathway pair activity score.

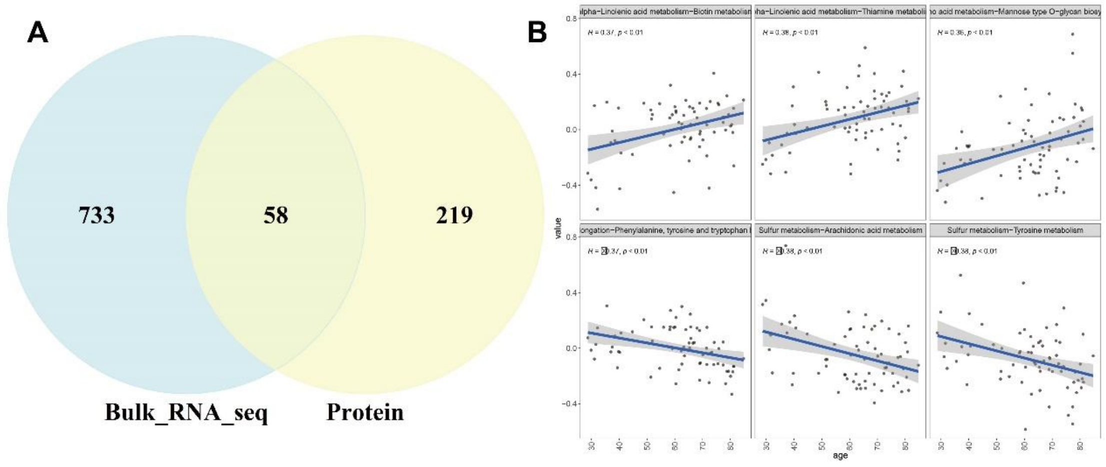

Figure S3. (A) the vein plot revealing the intersection significant metabolic switches based on the MMP^{3}C analysis using proteomics and transcriptomics datasets. **(B)** Pearson correlation analysis between age and metabolic pathway pair activity score calculated via GBM protein expression datasets.

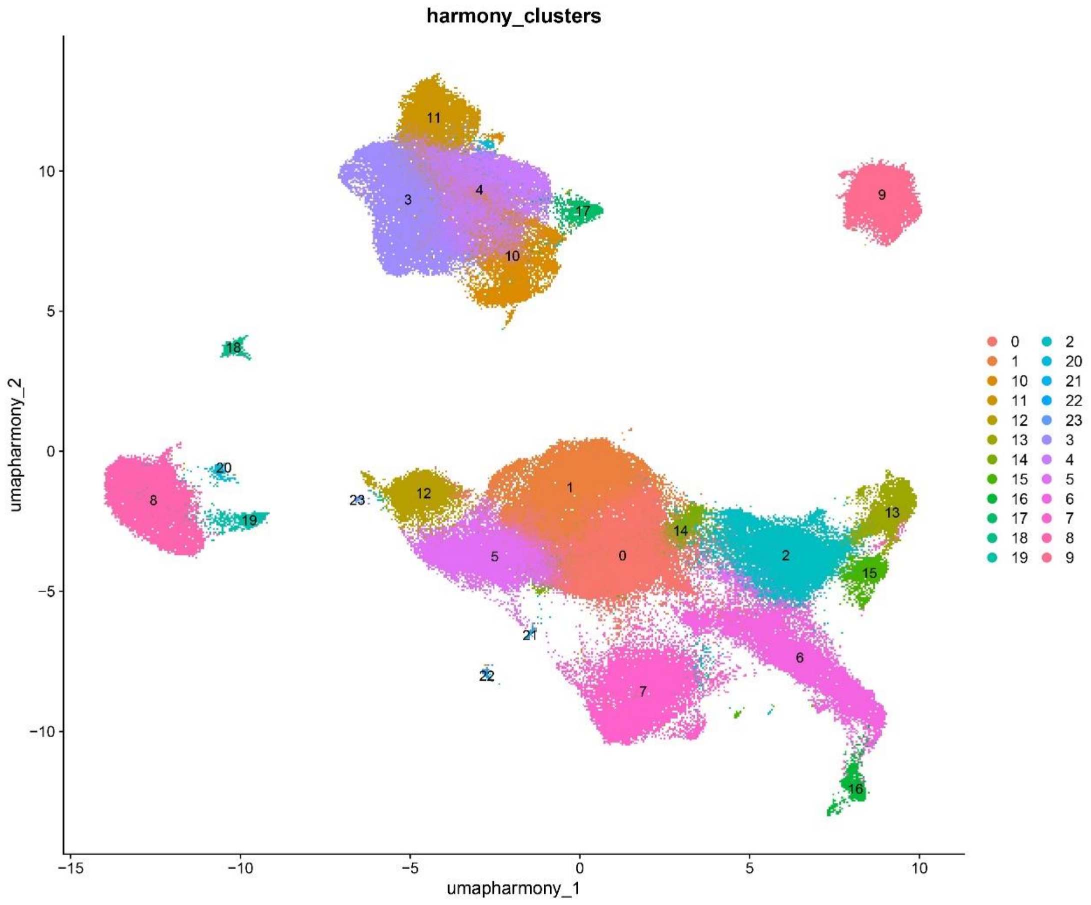

Figure S4. UMAP plot of 181,286 cells from 24 primary glioma patients displaying 24 main cell clusters.

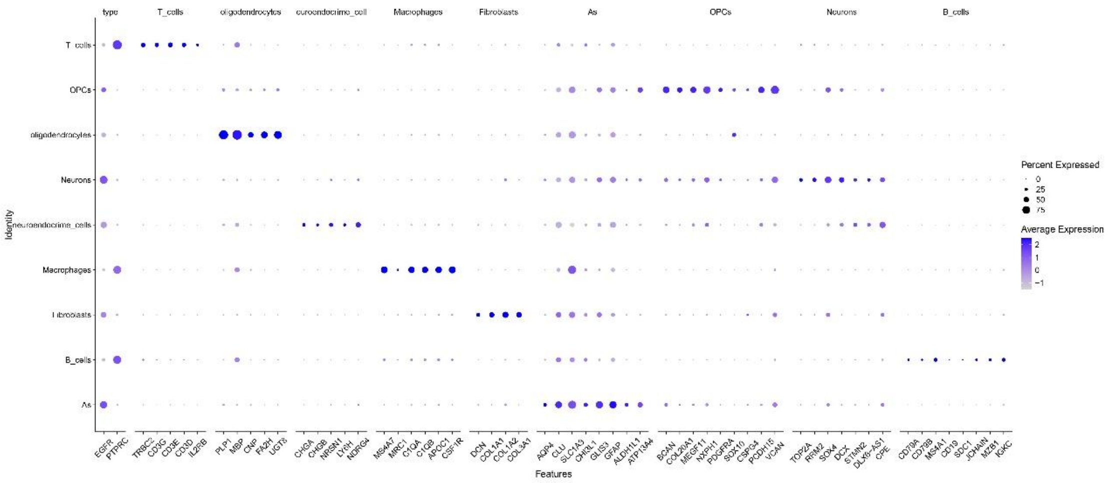

Figure S5. Dot plot of significant markers of each cell type in the tumor microenvironment.

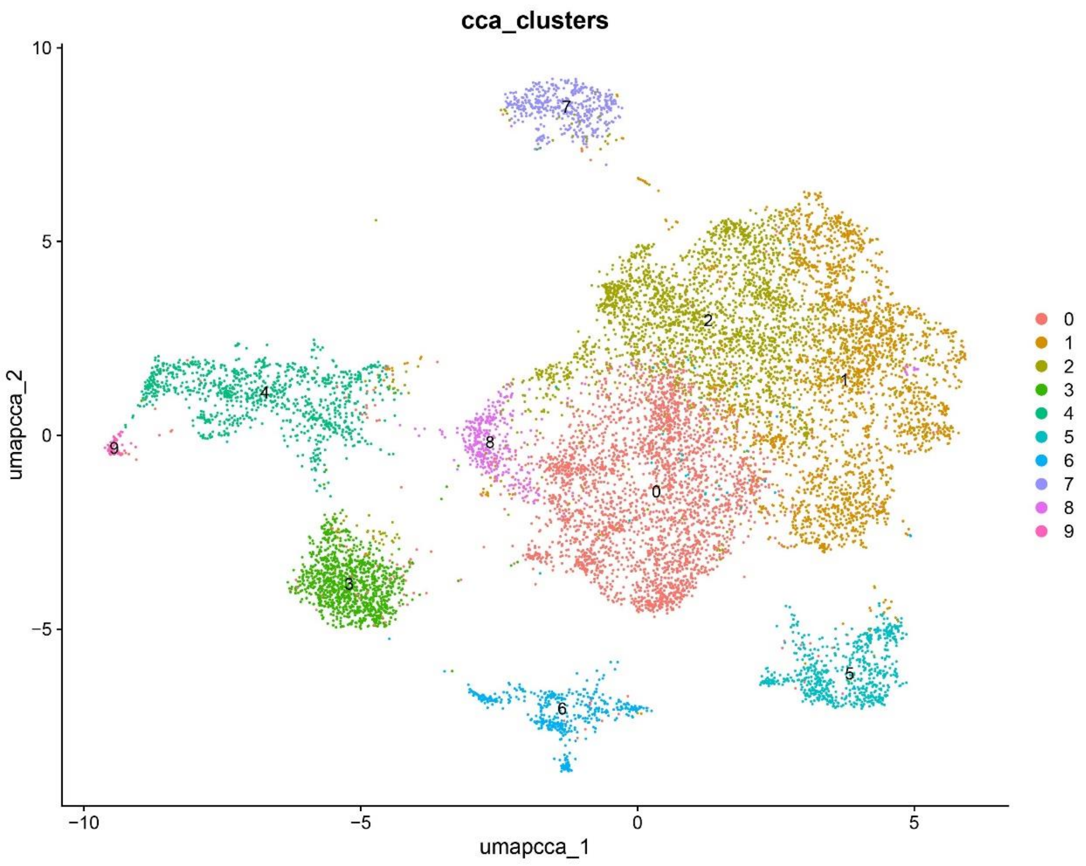

Figure S6. UMAP plot of all 12,144 T cells from primary glioma patients displaying 10 T cell clusters.

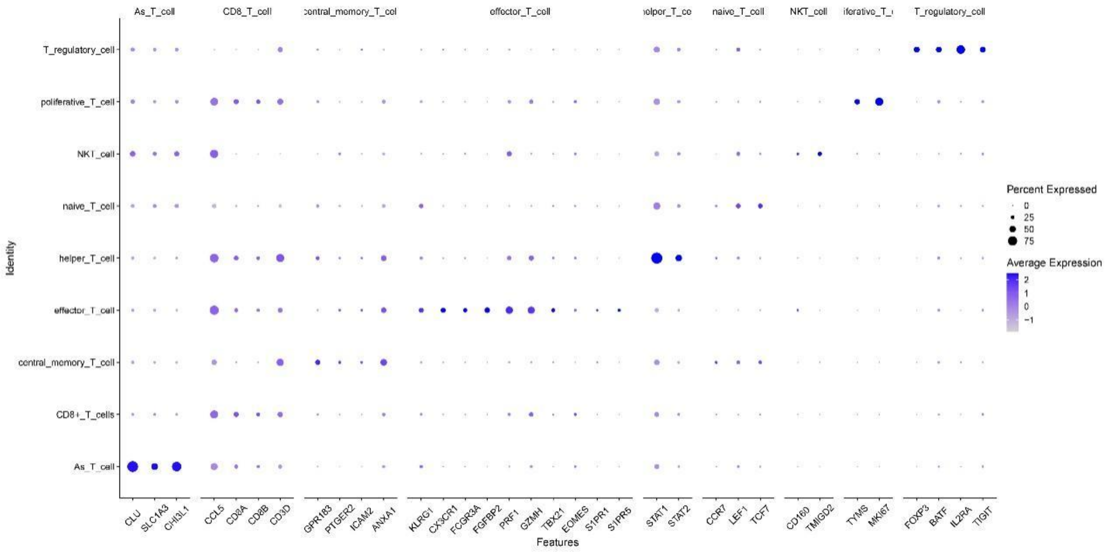

Figure S7. Dot plot of significant markers of each T cell type in the tumor immune microenvironment.

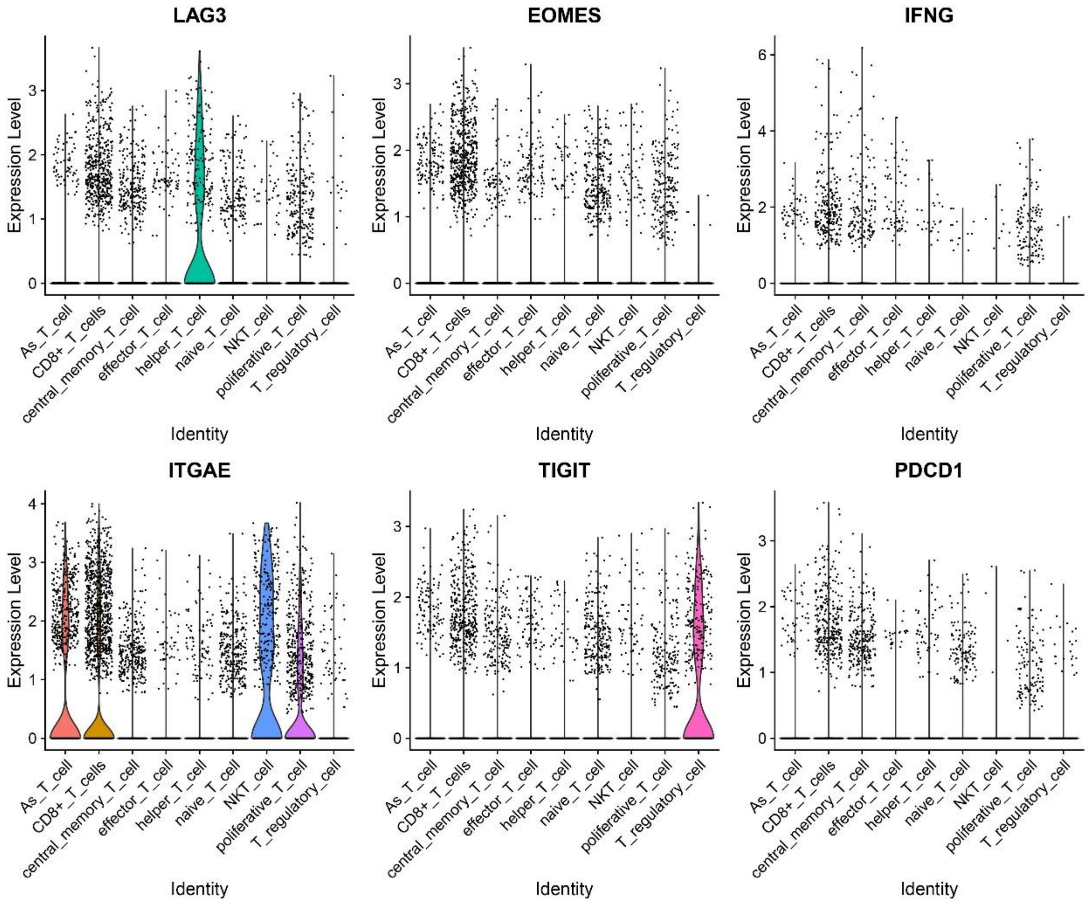

Figure S8. The violin plot of T cell exhausted markers in different cell subtypes in the trajectory analysis.

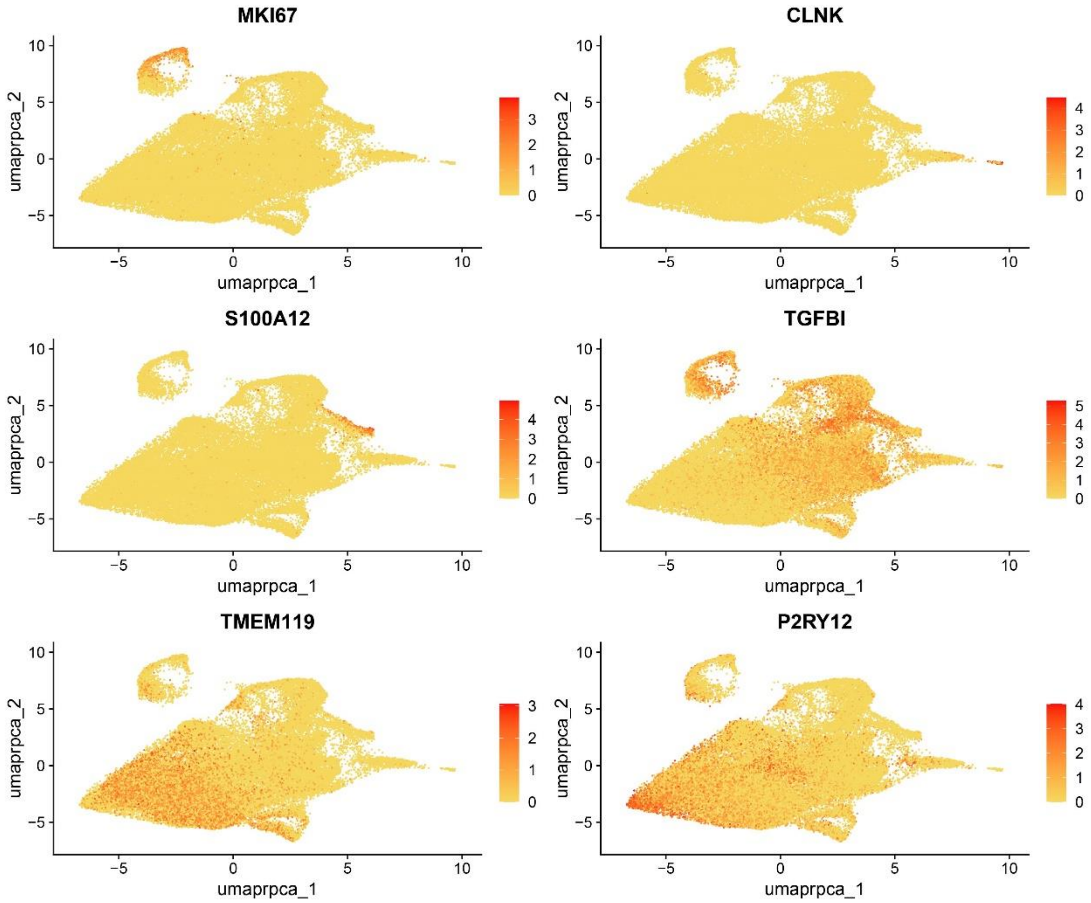

Figure S9. The UMAP clustering of the mRNA expression of significant marker genes of TAMs.

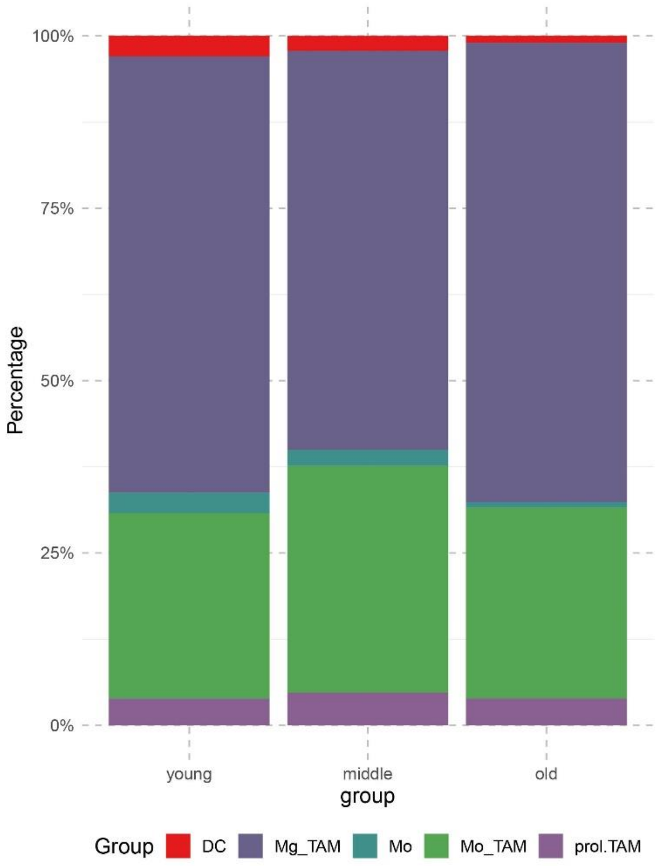

Figure S10. Percent bar plot unveiling the distribution of macrophages among age-related groups.

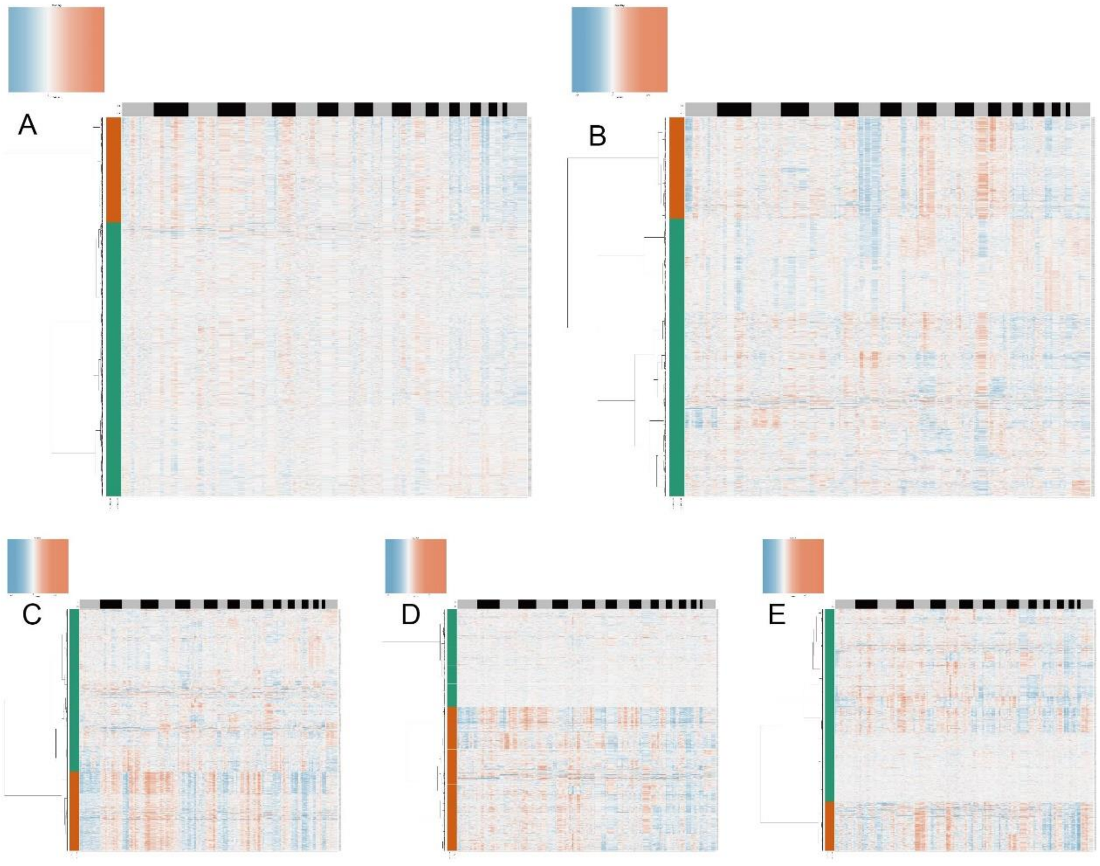

Figure S11. The CNV heatmap inferring to non-malignant and malignant cells from oligodendrocytes (A), oligodendrocyte progenitor cells (B), and astrocytes (C-E). Astrocytes were divided into three clusters for the run of CNV inferring due to memory limit.
